# Creation of an Evidence-Based Implementation Framework for Digital Health Technology in the Intensive Care Unit: Qualitative Study

**DOI:** 10.2196/22866

**Published:** 2022-04-08

**Authors:** Lina Katharina Mosch, Akira-Sebastian Poncette, Claudia Spies, Steffen Weber-Carstens, Monique Schieler, Henning Krampe, Felix Balzer

**Affiliations:** 1 Institute of Medical Informatics Charité – Universitätsmedizin Berlin Corporate Member of Freie Universität Berlin and Humboldt-Universität zu Berlin Berlin Germany; 2 Department of Anesthesiology and Intensive Care Medicine Charité – Universitätsmedizin Berlin Corporate Member of Freie Universität Berlin and Humboldt-Universität zu Berlin Berlin Germany

**Keywords:** digital health, patient monitoring, intensive care medicine, intensive care unit, technological innovation, user-centered, usability, implementation, implementation science, qualitative research, interview

## Abstract

**Background:**

Digital health technologies such as continuous remote monitoring and artificial intelligence–driven clinical decision support systems could improve clinical outcomes in intensive care medicine. However, comprehensive evidence and guidelines for the successful implementation of digital health technologies into specific clinical settings such as the intensive care unit (ICU) are scarce. We evaluated the implementation of a remote patient monitoring platform and derived a framework proposal for the implementation of digital health technology in an ICU.

**Objective:**

This study aims to investigate barriers and facilitators to the implementation of a remote patient monitoring technology and to develop a proposal for an implementation framework for digital health technology in the ICU.

**Methods:**

This study was conducted from May 2018 to March 2020 during the implementation of a tablet computer–based remote patient monitoring system. The system was installed in the ICU of a large German university hospital as a supplementary monitoring device. Following a hybrid qualitative approach with inductive and deductive elements, we used the Consolidated Framework for Implementation Research and the Expert Recommendations for Implementing Change to analyze the transcripts of 7 semistructured interviews with clinical ICU stakeholders and descriptive questionnaire data. The results of the qualitative analysis, together with the findings from informal meetings, field observations, and previous explorations, provided the basis for the derivation of the proposed framework.

**Results:**

This study revealed an insufficient implementation process due to lack of staff engagement and few perceived benefits from the novel solution. Further implementation barriers were the high staff presence and monitoring coverage in the ICU. The implementation framework includes strategies to be applied before and during implementation, targeting the implementation setting by involving all ICU stakeholders, assessing the intervention’s adaptability, facilitating the implementation process, and maintaining a vital feedback culture. Setting up a unit responsible for implementation, considering the guidance of an implementation advisor, and building on existing institutional capacities could improve the institutional context of implementation projects in the ICU.

**Conclusions:**

Implementation of digital health in the ICU should involve a thorough preimplementation assessment of the ICU’s need for innovation and its readiness to change, as well as an ongoing evaluation of the implementation conditions. Involvement of all stakeholders, transparent communication, and continuous feedback in an equal atmosphere are essential, but leadership roles must be clearly defined and competently filled. Our proposed framework may guide health care providers with concrete, evidence-based, and step-by-step recommendations for implementation practice, facilitating the introduction of digital health in intensive care.

**Trial Registration:**

ClinicalTrials.gov NCT03514173; https://clinicaltrials.gov/ct2/show/NCT03514173

## Introduction

### Background

In intensive care medicine, digital health technologies promise to improve outcomes by reducing the patients’ length of stay or preventing complications [1-3]. Continuous remote monitoring allows early detection of deterioration in intensive care unit (ICU) patients and therefore rapid therapeutic intervention [4]. Algorithms used in clinical decision support systems and early warning scores can analyze the large amounts of data generated by ICU monitoring devices to decrease ICU mortality and the risk of complications such as prescription errors [5,6]. Despite the potential, the digital transformation of health care is lagging in numerous countries for reasons that can be ascribed at every level of the health care system. At the macro level, weak national internet infrastructures, high market fragmentation, and lack of legal frameworks, financing models, and interoperability play a significant role [7-9]. At the meso and micro levels, cumbersome operation, high costs, lack of interoperability, information governance uncertainty, and organizational resistance block digital health technology implementation [10-13].

Implementation science, as an increasingly evolving discipline, has brought about the publication of numerous guidelines and recommendations for the implementation of digital health technologies in health care settings by various institutions and researchers [9,14-17]. However, still scarce is the evidence regarding meso- and micro-level implementation and the guidelines for the successful integration of digital health technologies into specific clinical settings [16,18-20]. Successful and sustainable implementation in health care requires a holistic concept to be followed, applying meaningful strategies at all levels [21-23]. In particular, the implementation processes of digital health tools in German ICUs are poorly explored, apart from the concept *tele-ICU*, which involves augmenting local ICU capacity with external expertise through video consultation, remote monitoring, and web-based access to patient data management systems [1,24,25].

Five domains are essential for the implementation of digital health in various health care settings: (1) the individual digital health technology (eg, remote patient monitoring systems), (2) the outer setting (eg, external regulations, laws, and patient needs), (3) the inner setting (eg, the direct implementation environment, social factors, networks, and communication), (4) the individual health professionals, and (5) the implementation process [11]. These domains were first outlined in the Consolidated Framework for Implementation Research (CFIR), a well-proven tool to evaluate the implementation of an intervention into health care settings [12,13,26-29]. Targeting the improvement of implementation performance, the Expert Recommendations for Implementing Change (ERIC) provide a comprehensive compilation of strategies to boost implementation in clinical practice [30,31]. The CFIR domains and ERIC strategies are coherent and synergistic and provide meaningful guidance for implementation researchers and practitioners; however, they require more use cases and documentation of applications in a specific context and setting. In addition, the present literature on implementation strategies for digital health technologies in health care settings and particularly the ICU is extensive and unstructured, and the strategies reported are often poorly conceived [20,32,33].

It is unclear whether the aforementioned barriers and facilitators to digital health implementation can be transferred into the ICU context, given that it is a very specific setting: multiple professional groups work together, many different technologies are already in place, and staff stress levels are also high because of critically ill patients requiring acute treatment, high alarm frequency, and staffing and capacity constraints [34-36]. Concrete implementation strategies for digital health technologies in intensive care settings are still lacking.

### Objectives

This study aims to (1) investigate barriers and facilitators to the implementation of a remote patient monitoring technology and (2) develop a proposal for an implementation framework for digital health technology in the ICU.

## Methods

### Overview

To assess the barriers and facilitators to implementing a remote patient monitoring system, we explored stakeholder perspectives using an abductive qualitative approach. This research design, combining inductive and deductive elements, included semistructured interviews with ICU leaders and key stakeholders in the implementation process, as well as field observations and regular feedback discussions within the research team. To develop the presented implementation framework for digital health technology in the ICU, we conducted a deductive analysis by matching the collected data to the CFIR and ERIC domains. Using the CFIR-ERIC mapping tool, we filtered out relevant strategies to improve implementation performance. In a final step, the strategies were ordered in a temporal sequence and visualized in a figure [37]. The Standards for Reporting Qualitative Research were consulted to report this research [38].

### Ethics Approval and Consent to Participate

The ethical approval for this study was granted by the Ethics Commission of the Charité–Universitätsmedizin Berlin (EA1/031/18). Participation in the survey was voluntary. Before the study, all participants provided their consent.

### Context and Technical Setup

We conducted this study with ICU staff from a German university hospital over the course of the implementation of the Virtual Patient Monitoring Platform Vital Sync (version 2.4; Medtronic plc). The device remotely monitored ICU patients from portable tablet computers at the hospital premises and was supplemental to the primary patient monitoring system, the IntelliVue patient monitoring system (MX800 software version M.00.03; MMS X2 software version H.15.41-M.00.04; Koninklijke Philips N.V.). The primary Philips IntelliVue monitoring system displayed the vital parameters on stationary touchscreen displays at the bedside and on a monitor at the central nurse station. COPRA (version 6; COPRA System GmbH) was used as the patient data management system (PDMS); however, no data transmission from the Vital Sync system to the primary monitoring system or PDMS occurred.

The remote monitoring system was installed between May 2018 and June 2019 in 50% (5/10) of the beds of the postanesthesia care unit, an ICU mainly for postoperative patients that need short-term intensive care treatment and monitoring. The system included 2 sensors (the pulse oximetry and the capnography) that registered peripheral capillary oxygen saturation, pulse rate, end-tidal carbon dioxide level, and respiratory rate at a frequency of 1 Hz. The vital parameters were displayed on a monitor at the central nurse station and were retrievable from 6 tablet computers (2 large 10.2“ iPad tablets [9th generation; Apple Inc], 2 iPad mini 4 tablets [Model A1550; Apple Inc], and 2 Surface Pro 4 laptops [Microsoft Corporation]). A 6-digit code protected the iPad access, and the data were accessible after logging into the Vital Sync website. A username and a password protected the access to the Microsoft Surfaces. Technical instructions of ICU staff (ie, physicians, nurses, and respiratory therapists) into the device were conducted over a period of 1 month. In addition, 2 workshops were conducted for hands-on training. Additional assistance was provided as needed. Further technical description and use of the software can be found elsewhere [39,40].

### Study Design and Research Team

**Figure 1:**
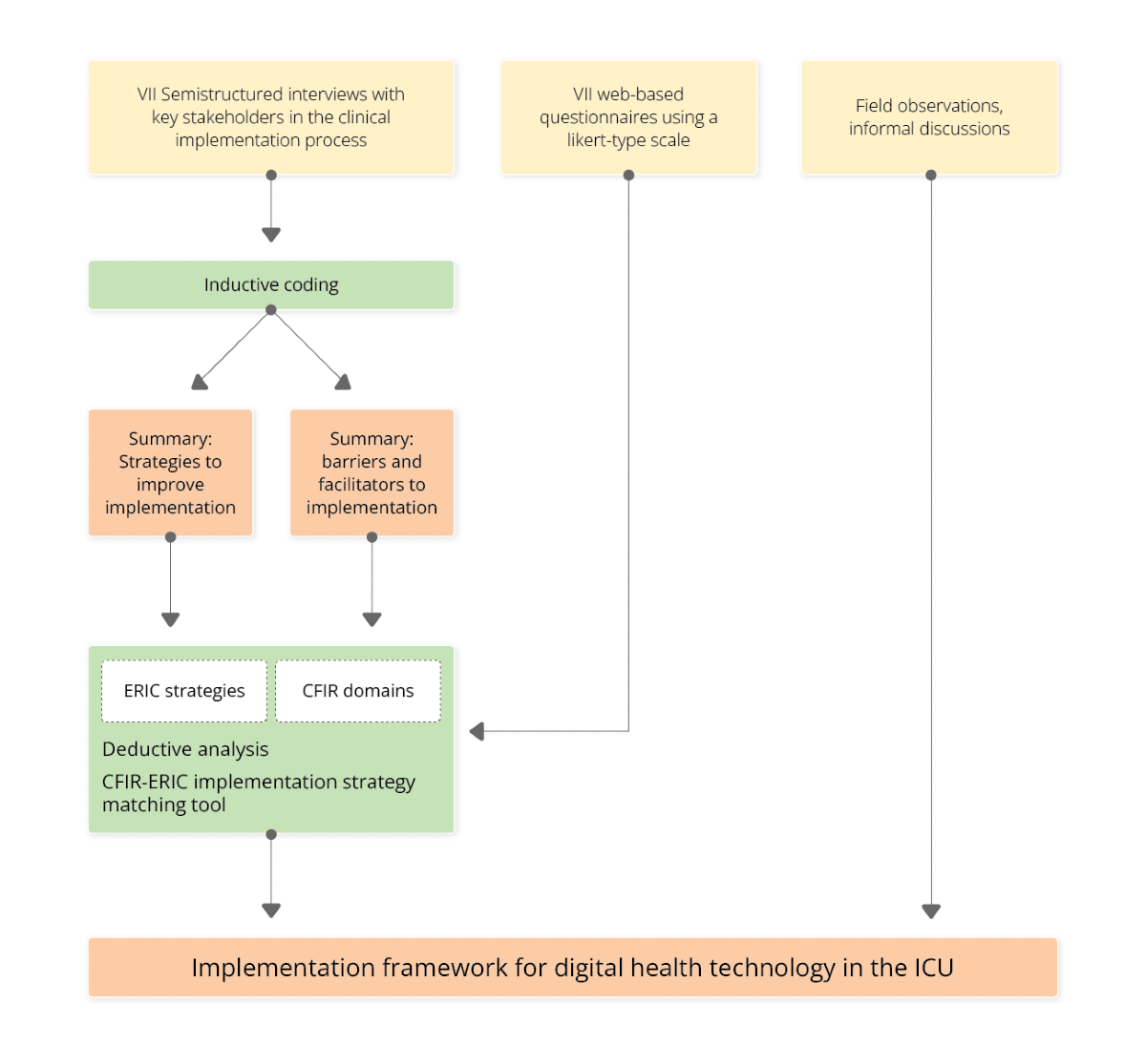
Overview of the data collection and analysis for the derivation of the proposed implementation framework for digital health technology in the ICU. CFIR: Consolidated Framework for Implementation Research; ERIC: Expert Recommendations for Implementing Change; ICU: intensive care unit.

This qualitative exploratory implementation study is based on an abductive research approach, as described by Dubois and Gadde [41] and Zainal [42]. The abductive approach of systematic combining (containing inductive and deductive analysis methods) specifies existing theories, refining them according to the individual case and context. We considered this approach essential to derive practical recommendations for the implementation of new technology in the ICU. The transcripts of 7 semistructured interviews and web-based questionnaires with key stakeholders in the clinical implementation process, the results of field observations and informal discussions among the research group, and findings from previous explorations in the context of the implementation were analyzed and applied to the CFIR domains and ERIC strategies to develop the proposed implementation framework (Figure 1) [43].

The research team consisted of an MD candidate (LKM); a postdoctoral researcher with a background in anesthesiology, intensive care medicine, digital health, and geriatrics (ASP); a professor for digital health, who is a consultant anesthesiologist and a computer scientist (FB); a psychologist (HK); a head nurse (MS); an ICU senior consultant (SWC); and the department’s head of staff (CS).

### Data Collection

Our data included interview transcripts and quantifiable results of a questionnaire with key stakeholders in the ICU, informal meetings and discussions among the research group, field observations, and the results of previous explorations [39,43]. The outer setting and manufacturer’s perspective were not part of this study because we could not evaluate these domains with the data given.

From June to November 2019, we conducted 7 semistructured interviews with ICU staff members, including 3 physicians, 3 nurses, and 1 respiratory therapist. We used purposive sampling with the aim of including all stakeholders who were closely involved in the implementation process and in leading positions in the ICU and presenting all professional groups. The identified study participants were key stakeholders (eg, head nurse, senior physician, and staff member with high working time in respective ICU) of the ICU and had closely experienced remote patient monitoring implementation, overseeing the implementation process, receiving feedback regarding the system from other staff members, and using the system in their own clinical practice.

The interview guideline was deduced on the findings of a previous study from our research group [43] and was oriented toward the categories of the CFIR (Multimedia Appendix 1 [44]). Pilot interviews with associated intensive care physicians did not alter the questions. The interviews were performed either before or after patient care and were recorded and transcribed verbatim.

The semistructured interview guideline included web-based questionnaires containing 47 items and a technology commitment scale [44]. We conducted face-to-face pilot testing with ICU staff with a focus on clarity, relevance, and order of the items. We used a 5-point Likert-type scale as an ordinal response format, with the options *not correct at all*, *not quite correct*, *partly correct*, *quite correct*, and *completely correct*. The study data were collected and managed using Research Electronic Data Capture (REDCap) tools hosted at Charité–Universitätsmedizin Berlin [45,46]. Data resulting from the questionnaire responses were collected in an overview table.

To gain auditability and enhance reflexivity in the research process, informal meetings and discussions among the research group and field observations occurred from the start of the implementation in May 2018 until March 2020. These methods helped gain a more objective perspective and minimize potential biases that naturally arise when using a qualitative research approach, as described by Noble and Smith [47]. Results of the field research were published by Poncette et al [39].

### Data Analysis

For qualitative analysis, we applied a hybrid approach combining inductive and deductive coding elements, as described by Fereday and Muir-Cochrane [48].

First, the interview transcripts were analyzed using a thematic analysis approach, applying an inductive coding process, meaning that themes and codes were iteratively developed and applied to all transcripts [49]. The resulting content of the codes was summarized to obtain the main findings and serve as the basis for the deductive analysis, as described by Crabtree and Miller [50].

Second, for deductive analysis, we used as code system templates the CFIR domains and ERIC strategies, which were grouped into 9 clusters [30,31]. Summaries from the inductive analysis and the findings of the questionnaires were coded according to templates (Multimedia Appendices 2 and 3). That is, data from the web-based questionnaires were not analyzed with quantitative methods. Specifically, the CFIR template was used to analyze the summaries regarding implementation performance, whereas the ERIC strategies served as a template for analyzing the summaries of staff’s suggestions on implementation process improvements. All coding was performed using the MaxQDA 2020 qualitative data analysis software [51].

Finally, the proposal for an implementing framework for digital health technology in the ICU was derived from the results of the CFIR- and ERIC-guided analyses. The CFIR-ERIC Implementation Strategy Matching Tool supported the prioritization of the derived recommendations [52]. Findings from the informal meetings, discussions, and field observations supported in situating the results and the interview suggestions in the context of implementation and in supplementing objective characteristics. We ordered the findings into a temporal perspective.

## Results

### Overview

Inductive analysis of the interview transcripts revealed the two major categories *perceived performance of the implementation* and *perceived factors improving implementation*, which contained 4 and 3 subtopics, respectively. According to the interviewed stakeholders, the remote patient monitoring system’s implementation was insufficient owing to a lack of staff engagement in the process and little perceived benefit from the novel solution in its current version. Factors suggested improving implementation were targeting staff training, features of the technology itself, and implementation setting.

Deductive coding revealed four major CFIR domains: *intervention characteristics*, *inner setting*, *individual characteristics*, and *process*. Regarding perceived factors improving implementation, seven clusters of the ERIC framework were mapped: *use evaluative and iterative strategies*, *provide interactive assistance*, *adapt and tailor to context*, *develop stakeholder interrelationships*, *train and educate stakeholders*, *support clinicians*, and *change infrastructure*.

### Implementation Process

#### Staff Involvement and Training

The interviewees identified staff involvement and training as being more targeted toward nursing staff, although they were not in charge of the implementation project. According to the interviewed stakeholders, staff members of all professional groups lacked a feeling of responsibility to continuously apply the remote patient monitoring system. In addition, the staff was unable to identify a leading member in charge of the implementation process and longed for more regular staff training and information sessions. Interviewees reported that opinion leaders’ communication created a negative peer pressure not to use the system.

Interviewees said that they felt well informed about the project initially; however, the information flow decreased equally. Training did not reach all staff members because of a complex shift system and a big pool of staff for 2 ICUs, whereas the system was implemented only at 50% (5/10) of bedsides on 1 ICU. Staff perceived the system as an imposition from outside the ICU and felt that it did not have any influence on the implementation.

#### Additional Benefit

Staff did not perceive the system’s added value as high for four reasons: First, the ICU already had a monitoring system offering remote functions (eg, displaying vital parameters of different patients on all bedside monitors), although it did not offer a portable monitoring device. However, according to interviewees, this made an additional system superfluous. Second, the high staff presence in the ICU decreased the need to remotely monitor patients. Third, high patient turnover in the ICU was associated with frequent connecting and disconnecting of patients to and from the system, resulting in an increased workload for nurses. Fourth, remotely monitoring patients while being on a different ward or performing a clinical intervention would make a necessary immediate reaction to an alarm impossible.

#### Intervention Features

Interviewees highlighted that the limited number of vital parameters monitored by the system was not sufficient to satisfactorily evaluate the patient’s condition. Furthermore, the system’s dependency on a stable wireless network connection raised concerns. Interviewees perceived the tablet as too large and inconvenient to use and carry in the tunic pockets. Finally, the device would not allow patients’ monitoring during their transportation.

#### Attitude of Staff

**Figure 2:**
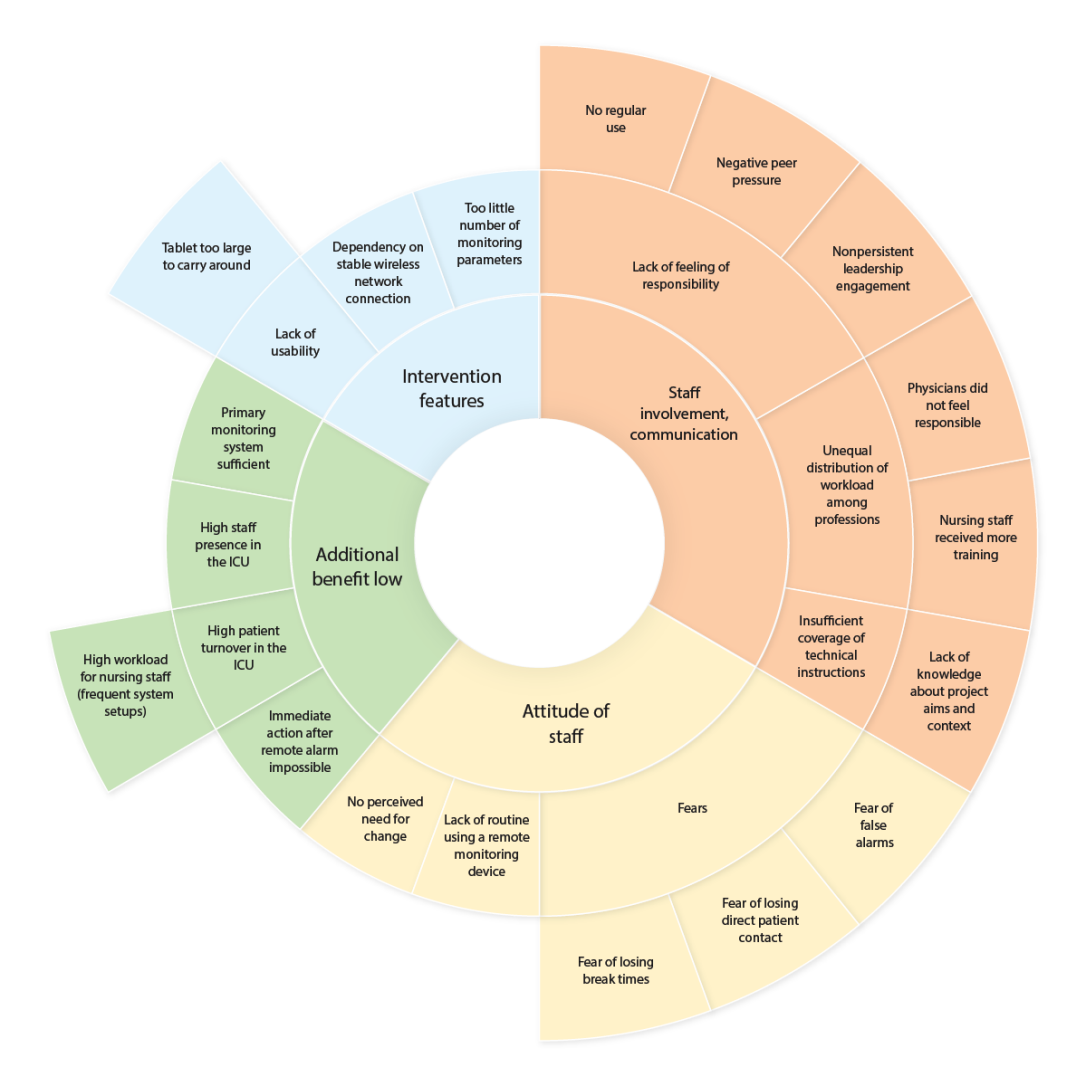
Implementation performance: 4 major categories were identified (inner ring), divided into themes (middle ring), and further specified (outer ring). ICU: intensive care unit.

Interviewees said that they were satisfied with the current monitoring system and did not see the need for a change. ICU staff did not use the system because they lacked the habit and routine of using a remote patient monitoring technology. They were afraid of losing break times when applying the system and of an increased workload (eg, system setup). They feared that reduced patient contact and false alarms might increase stress levels and endanger patient safety. Overall, the staff saw no additional benefit in the technology. Figure 2 presents an overview of the factors influencing the implementation process from the perspective of interviewed staff members.

### Mapping of CFIR Domains

The summaries of the staff interview transcripts and descriptive data from the questionnaire responses were coded and assigned to four major domains of the CFIR: intervention characteristics, inner setting, individual characteristics, and process (Textbox 1 and Multimedia Appendix 2 [44]).

**Mapped Consolidated Framework for Implementation Research domains and subdomains.**

**Intervention characteristics**

- Intervention source
- Evidence strength and quality
- Relative advantage
- Adaptability
- Trialability
- Complexity

**Inner setting**

- Structural characteristics
- Networks and communication
- Implementation climate: tension for change, compatibility, relative priority, and learning climate
- Implementation readiness: leadership engagement and access to information

**Individual characteristics**

- Knowledge and beliefs about the intervention
- Self-efficacy
- Individual stage of change

**Process**

- Planning
- Engaging: opinion leaders and formally appointed implementation leaders
- Executing

### Strategies to Improve Implementation

#### Staff Engagement and Communication

According to the interviewed stakeholders, persistent leadership engagement and nomination of specific responsible persons for the implementation process were essential, especially in a busy environment such as the ICU. Staff training should be conducted continuously and was particularly critical in the early implementation stages. The quality of instructions was considered essential to influence the staff’s opinion toward the implementation. Feedback discussions with staff, project leaders, and a well-functioning team would increase staff engagement. Communication of the project should be encouraging and motivating.

#### Setting

It was reported that equipping all beds in the ward with the technology and all staff members with portable monitoring devices would increase the implementation performance. A normal or intermediate care unit (IMCU) could be more suitable for a remote patient monitoring technology owing to a lower staff presence and scarcer technical facilities. Interviewees suggested that patients with a relatively weak indication for admission to the ICU could be admitted to a normal ward or IMCU and be monitored remotely. The implementation of technology concerning ICU patients would be more straightforward in a ward with more extended patient stays, as short stays imply more work to install the system.

#### Intervention Features

**Figure 3:**
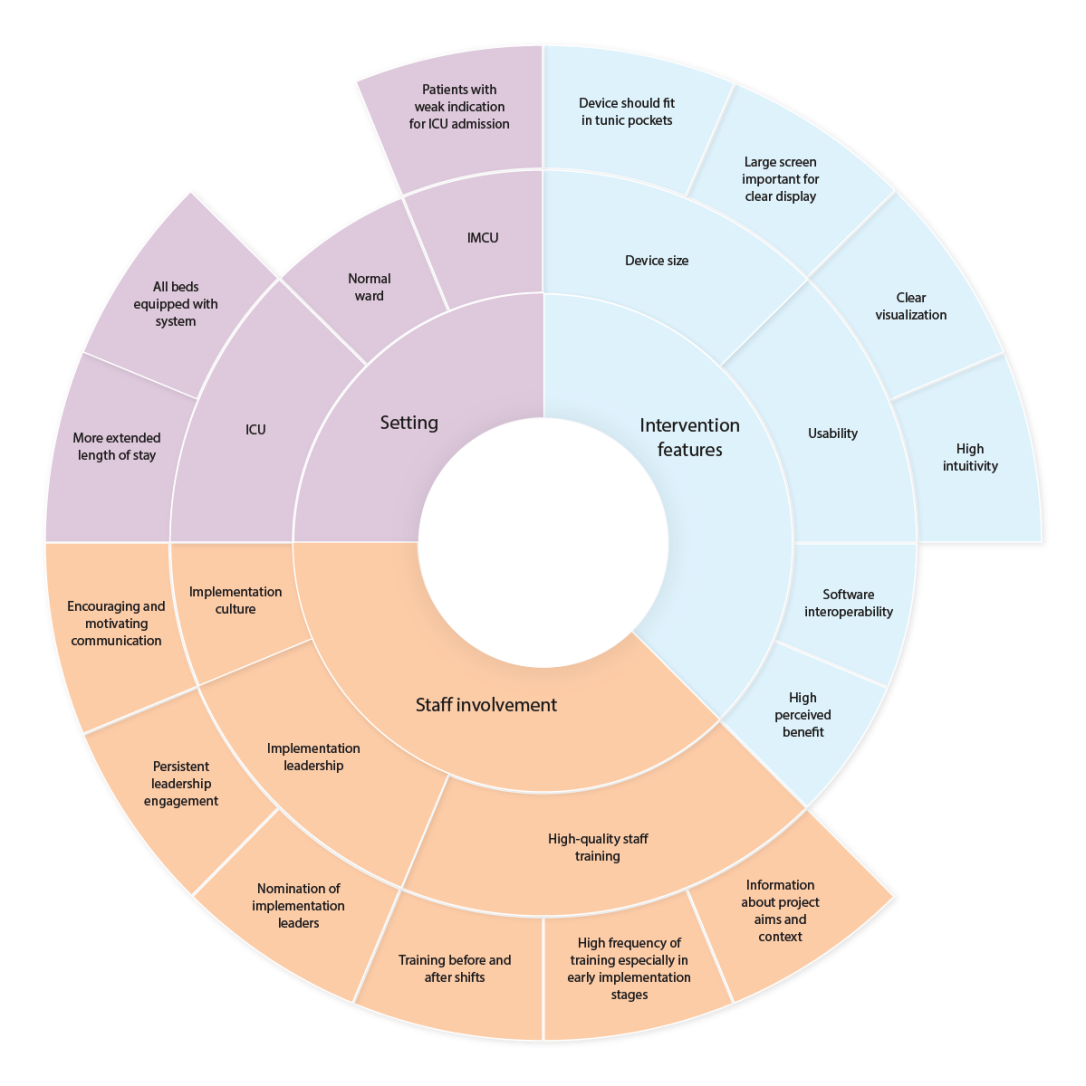
Perceived factors improving implementation: 3 categories were identified (inner ring), divided into subcategories (middle ring), and enriched with concrete suggestions (outer ring). ICU: intensive care unit; IMCU: intermediate care unit.

High intuitiveness would be crucial for effective implementation, as stated by the interviewees. A monitoring solution without cables would increase usability and perceived benefit. Opinions on the device size varied; a clear visualization needs a large screen, but interviewees favored a device that fits into the pocket of a tunic. Software interoperability with other devices (eg, the respirator or the PDMS) would be essential. Figure 3 presents an overview of the strategies to improve the implementation of digital health technologies according to the interviewed staff members.

### Mapping of ERIC Strategies

Of the 73 ERIC strategies, 19 (26%) were mapped to the summary segments concerning staff suggestions for implementation and quantifiable questionnaire responses (Textbox 2 and Multimedia Appendix 3). The segments were assigned to 78% (7/9) of the clusters of the ERIC framework.

**Mapped Expert Recommendations for Implementing Change clusters and strategies.**

**Use evaluative and iterative strategies**

- Purposely re-examine the implementation
- Develop a formal implementation blueprint
- Audit and provide feedback

**Provide interactive assistance**

- Facilitation
- Provide clinical supervision

**Adapt and tailor to context**

- Promote adaptability

**Develop stakeholder interrelationships**

- Identify and prepare champions
- Organize clinician implementation team meetings
- Recruit, designate, and train for leadership
- Inform local opinion leaders
- Model and simulate change
- Involve executive boards

**Train and educate stakeholders**

- Conduct ongoing training
- Make training dynamic
- Use train-the-trainer strategies
- Conduct educational meetings

**Support clinicians**

- Facilitate relay of clinical data to providers
- Remind clinicians

**Change infrastructure**

- Change physical structure and equipment
- Change service sites

### Proposal for an Implementation Framework for Digital Health Technology in the ICU

**Figure 4:**
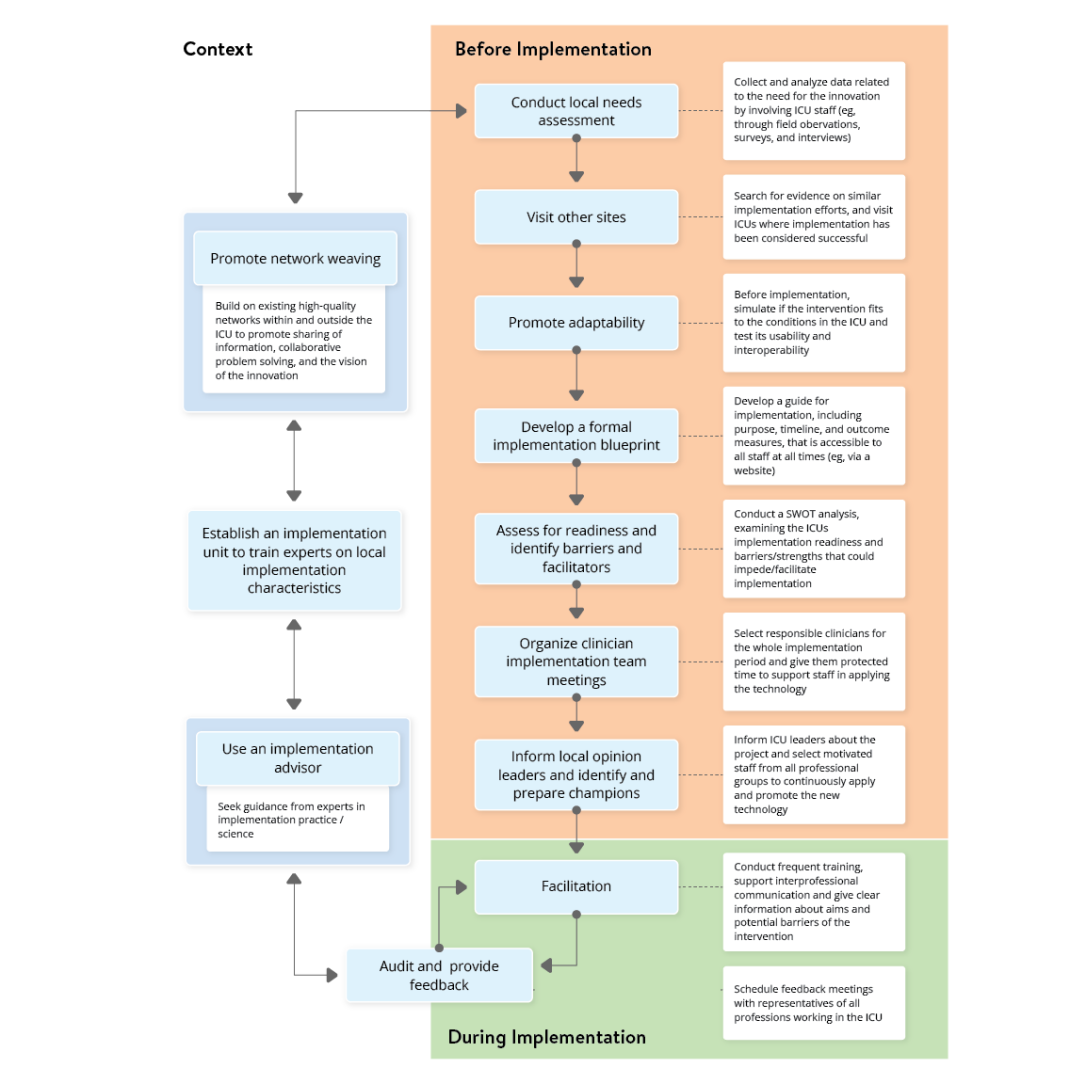
Strategies resulting from the CFIR-ERIC Implementation Strategy Matching Tool and the mapping of staff suggestions for improving implementation to the ERIC strategies before (orange) and during (green) implementation and in the general context of the implementation (yellow). CFIR: Consolidated Framework for Implementation Research; ERIC: Expert Recommendations for Implementing Change; ICU: intensive care unit; SWOT: strengths, weaknesses, opportunities, and threats [52].

The developed implementation framework includes 11 recommendations derived from ERIC strategies belonging to 4 clusters of the ERIC framework. A temporal perspective was added, and recommendations were specified to the ICU environment (Figure 4). Our recommendations are targeted toward hospital administrations, leading clinicians in the ICU, and implementation researchers—individuals responsible for the implementation process of new digital health technology in the ICU. Before implementation, 7 strategies, such as *conduct local needs assessment, visit other sites,* or *promote adaptability*, should be completed. During the implementation process, we recommend applying the ERIC strategies *facilitation* and *audit and provide feedback* continuously. The strategies *promote network weaving* and *use an implementation advisor* should optimize the implementation setting’s context. Optimally, an implementation unit with experts for the local implementation characteristics should be established. Several factors influence the choice of the time to start the implementation process, and an implementation advisor should be consulted to adapt these factors to the context and local needs. Regular feedback by ICU staff regarding the implementation process, illustrated in Figure 4, through the feedback loop can lead to a further need for innovation and ideas to implement digital health technologies. The implementation is a circular process; therefore, we did not include an *after implementation* phase. Continuous re-evaluation triggers a new entry into the implementation strategy and thus leads to a sustainable implementation environment that is always adapted to new needs.

## Discussion

### Principal Findings

Taking the example of a remote patient monitoring system, this study confirmed critical barriers to the implementation of new digital health technologies in the intensive care setting [11,13,53]. The proposed implementation framework for digital health technology in the ICU includes practical strategies to overcome these barriers while using facilitators from the ERIC clusters that can be applied before and during implementation and in the general context of an implementation.

Before implementation and in the general context, sharing use cases and building upon existing best practices are crucial strategies to adapt and choose the technology that best fits the local settings (ie, *visit other sites*, *promote network weaving*, and *use an implementation advisor*) [13,21]. Initiators of an implementation project should lay out its details, aim, and context before implementation (*develop a formal implementation blueprint)*. Transferable discoveries from these strategies and the strategies we propose to be applied before implementation (*promote adaptability*, *conduct local needs assessment*, *assess for readiness*, and *identify barriers and facilitators*) could be used to improve the adaptability and needs orientation of the intervention. Adaptability and user-centered design have been identified as key facilitators of digital health implementation in other settings [11,53,54]. To create the respective conditions, developers and providers of digital health technologies should actively participate in the implementation processes by taking advantage of the valuable feedback from clinical stakeholders and adapting their products in the spirit of user-centered design [55-57]. Therefore, our proposed implementation framework suggests several strategies to enhance the involvement of clinical stakeholders directly (*organize clinician implementation team meetings*, *inform local opinion leaders*, and *identify and prepare champions*), in line with the proposed strategies for other implementation settings [58,59].

During implementation, ensuring a transparent communication of the project’s aim and context (*audit and provide feedback*) is as critical as an effective *facilitation* to improve staff involvement and to promote and sustain implementation.

Sustainable implementation practice means to include the aforementioned aims and strategies in the general context of implementation practice. We propose the strategies *use an implementation advisor* and e*stablish an implementation unit* to improve the implementation environment and the local conditions for a fast, efficient, and sustainable implementation of technology that focuses on the needs of users and patients and adds value. These processes should always be re-evaluated to readapt interventions following the changing needs [58,60,61].

### Implementing Technology in the ICU

For decades, the ICU has been equipped with high technology to support staff with continuous monitoring of patients’ vital signs, application of medication, documentation (eg, PDMS), or diagnostics (eg, ultrasound and bronchoscopy). However, the implementation of innovative technology in a demanding and hectic environment such as the ICU is a challenge [62]. This has been prominently shown by various projects, more recently, through the rise of telemedicine in the ICU [63], necessitating frameworks for the implementation of such endeavors.

Reported digital health implementation efforts in the ICU rarely involved the use of developed implementation frameworks [64]. This could be due to a lack of both implementation expert consultation and framework transferability into clinical routine. Current frameworks for the design and implementation of digital health technologies are based on best practices and, if evidence-based, need to be validated [30,65]. Our study provides an explicit approach to target implementation challenges and optimize innovation flows and adaptability in the complex environment of an ICU. Further optimization by saturating theories with practical experiences from clinical translation is crucial for the development of a scalable and agile framework for the implementation of digital health technology in the ICU.

### Internet of Things, Interoperability, and Data Security

Especially in ICU settings, where various technical devices continuously generate data, the amount of data that can be analyzed and processed is growing rapidly [66-68]. With growing amounts of data to analyze and process, the adoption of the Internet of Things (IoT) in health care is a promising approach to alleviating issues such as high staff stress levels, alarm fatigue, and even medical errors [69,70]. ICUs, in particular, use many different end devices that could be integrated into a fog-, edge-, or cloud-based IoT network for fast and efficient data processing [71,72].

To enhance the capacities of cloud systems, interoperability has become increasingly important, especially in relation to IoT infrastructures [73,74]. Holistically implemented, interoperable technologies could alleviate the burden on staff by reducing documentation time, and easier data retrieval can facilitate therapy and diagnosis [75]. The lack of interoperability of the remote patient monitoring system may have presented a barrier to its implementation. Consistent with findings from other research [55,76], our results show that health care staff support the implementation of interoperable, intelligent monitoring interfaces.

When harnessing the potential of interoperable IoT networks and implementing them in health care settings, a secure and reliable IT infrastructure is required [77,78]. Cybersecurity in health care organizations should be fostered through the definition of cybersecurity duties, sufficient funding, and the application of state-of-the-art measures to reduce the risk of cyberattacks [79,80]. For instance, blockchain technology combined with IoT-enabled smart devices using interoperable fog/edge and cloud computing networks can enable secure, instantaneous data transmission and processing while reducing costs and network delays [70,71].

### Implementation Units

With aforementioned promised benefits, health care providers will experience the need to implement new digital health technology into their infrastructures in the decades to come [63,81-83]. They have to be abreast of the latest digital health technologies to select the appropriate technology for the specific area of application and to plan and execute the implementation process, requiring an effective and efficient approach to implementation.

The question arises as to which staff position is responsible for overseeing, evaluating, and adapting recent evidence and strategies in implementation science to the local context. As suggested, internal and external implementation experts should be involved as early as possible [30]. With the introduction of a unit for implementation as a central starting point for any implementation project, resources for redundant project planning or ineffective implementation could be spared and invested elsewhere. The extent to which these units will be involved in the ICU design, for example, should be assessed individually. Beyond the consultation and proposal regarding innovations, such a unit could assess the usability of devices and the adaptability of the intervention before procurement [84] or foster exnovation and deimplementation of outdated or useless technology.

### Implementation Frameworks

Implementation science is a young discipline that has developed over the last 2 to 3 decades [85]. Nonetheless, numerous implementation frameworks, either for specific health care settings or for general guidelines, have been published during this period [26,64,86-88]. Other implementation frameworks and strategies for health care are nonspecific in terms of either the intervention targeted [26,64], as they refer to evidence-based practices [89], or technology [90-92]. Looking at intensive care medicine, implementation frameworks are widely limited to the implementation of evidence-based practices [93,94]. Explicit guidelines for the implementation of novel digital health technologies in the special ICU environments are lacking.

The implementation framework at hand was developed through an interdisciplinary approach, is specific to the ICU setting, and considers relevant particularities in terms of digital health technology implementation.

### Limitations

The research team was only able to obtain a limited view of the entire implementation project. The decision to implement the system was made before the study began, which prevented conducting front-end exploration of the implementation setting or evaluation of the external setting and vendor perspective. It was not possible to pursue a user-centered design and implementation in this specific context. However, our study provides valuable insights into the process of implementing digital health technology in the ICU and highlights important application strategies while planning an implementation project. In particular, we identified explicit pitfalls for implementation processes in the specific clinical environment of an ICU and solutions to overcome them.

The interpretation of the results should consider that the CFIR-ERIC mapping tool needs further validation and evidence. Thus, the mapping of strategies to the major barriers might not reflect the best strategy to tackle the respective barrier. We sought to overcome this limitation through profound discussions at meetings within the research team, extensive field research, and analysis of suggestions from staff to improve the implementation performance.

A limitation to the study’s scope is that the ERIC strategies do not include changes in intervention characteristics, which would be essential when aiming to improve implementation performance in a user-centered design. ERIC only covers the last stages of implementation (planning and executing the implementation of the finalized intervention) but does not include the readaptation of the intervention as part of the development process.

Finally, the fact that every ICU has unique structural and sociotechnical features, as well as the number of interviewees, could limit the general validity of derived findings. As we investigated an explicit use case in an ICU, potential interviewees were limited because we identified and interviewed the key stakeholders throughout the study. This study depicts an implementation project in intensive care medicine that is close to the standard practice in Germany, where implementation science is still an evolving discipline. However, it is specific to the setting in which it was conducted (ICU, country, and health system), and translation of our findings to other contexts is limited and should be done with these specificities in mind. In terms of continuous reassessment, our proposed framework may need further validation and evaluation in ICU or IMCU settings to fully realize its potential for optimization of implementing digital technologies.

### Conclusions

We propose an implementation framework for digital technology in the ICU, which entails practical and evidence-based strategies to improve the implementation process. The ICU provides an exceptional setting for the introduction of digital health technology: the stress level of staff is high, and the ICU team is composed of multiple different professions using the same technologies.

The proposed framework outlines strategies to be applied before and during implementation and in the general context of implementation. Before implementation, the need for innovation and potential interventions should be carefully assessed by involving all clinical stakeholders with clear implementation leadership. Interventions should be needs-oriented, user-centered, and adaptable to changing circumstances. During implementation, a clinical implementation team should ensure transparent, inclusive, and motivating staff communication regarding the project and continuous feedback through local opinion leaders and champions. To ensure efficient management of resources and time, we recommend optimizing the general context of implementation practice in the ICU and the health care institution by involving an implementation advisor, ideally in consultation with an implementation unit of the same institution. Our proposed framework should encourage health care institutions to implement modern digital technology in ICUs and facilitate clinicians and implementation advisors in the practical execution of implementation projects in ICU settings.

## Appendix

Multimedia Appendix 1 Interview guideline.

Multimedia Appendix 2 Mapping of Consolidated Framework for Implementation Research domains to summaries of codes concerning implementation performance.

Multimedia Appendix 3 Expert Recommendations for Implementing Change strategies mapped to summaries of codes concerning staff suggestions for improving implementation performance.

## Abbreviations

CFIR: Consolidated Framework for Implementation Research
ERIC: Expert Recommendations for Implementing Change
ICU: intensive care unit
IMCU: intermediate care unit
IoT: Internet of Things
PDMS: patient data management system
REDCap: Research Electronic Data Capture

## Data Availability

The data sets generated and analyzed during this study are not publicly available because of data privacy; however, they are available from the corresponding author (FB) upon reasonable request.

## References

[1] Kumar S, Merchant S, Reynolds R (2013). Tele-ICU: efficacy and cost-effectiveness of remotely managing critical care. Perspect Health Inf Manag.

[2] Hawkins HA, Lilly CM, Kaster DA, Groves Jr RH, Khurana H (2016). ICU telemedicine comanagement methods and length of stay. Chest.

[3] Meyer A, Zverinski D, Pfahringer B, Kempfert J, Kuehne T, Sündermann SH, Stamm C, Hofmann T, Falk V, Eickhoff C (2018). Machine learning for real-time prediction of complications in critical care: a retrospective study. Lancet Respir Med.

[4] Khanna AK, Hoppe P, Saugel B (2019). Automated continuous noninvasive ward monitoring: future directions and challenges. Crit Care.

[5] Escobar GJ, Liu VX, Schuler A, Lawson B, Greene JD, Kipnis P (2020). Automated identification of adults at risk for in-hospital clinical deterioration. N Engl J Med.

[6] Prgomet M, Li L, Niazkhani Z, Georgiou A, Westbrook JI (2017). Impact of commercial computerized provider order entry (CPOE) and clinical decision support systems (CDSSs) on medication errors, length of stay, and mortality in intensive care units: a systematic review and meta-analysis. J Am Med Inform Assoc.

[7] Thiel R, Deimel L, Schmidtmann D, Piesche K, Hüsing T, Rennoch J, Stroetmann V, Stroetmann K (2018). #SmartHealthSystems - international comparison of digital strategies. Bertelsmann Stiftung.

[8] Bughin J, Hazan E, Labaye E, Manyika J, Dahlström P, Ramaswamy S, de Billy CC (2016). Digital Europe: realizing the continent's potential. McKinsey Global Institute.

[9] (2018). Communication on enabling the digital transformation of health and care in the Digital Single Market; empowering citizens and building a healthier society. European Commission.

[10] Wachter RM (2016). Making IT Work: harnessing the power of health information technology to improve care in England. British Association Of Social Workers.

[11] Ross J, Stevenson F, Lau R, Murray E (2016). Factors that influence the implementation of e-health: a systematic review of systematic reviews (an update). Implement Sci.

[12] Marcial LH, Johnston DS, Shapiro MR, Jacobs SR, Blumenfeld B, Rojas Smith L (2019). A qualitative framework-based evaluation of radiology clinical decision support initiatives: eliciting key factors to physician adoption in implementation. JAMIA Open.

[13] Lennon MR, Bouamrane MM, Devlin AM, O'Connor S, O'Donnell C, Chetty U, Agbakoba R, Bikker A, Grieve E, Finch T, Watson N, Wyke S, Mair FS (2017). Readiness for delivering digital health at scale: lessons from a longitudinal qualitative evaluation of a national digital health innovation program in the United Kingdom. J Med Internet Res.

[14] (2019). WHO guideline: recommendations on digital interventions for health system strengthening. World Health Organization.

[15] Expert Panel on effective ways of investing in Health (2019). Assessing the impact of digital transformation of health services. Publications Office of the European Union.

[16] World Health Organization, International Telecommunication Union (2012). National eHealth strategy toolkit. World Health Organization.

[17] Balas EA, Chapman WW (2018). Road map for diffusion of innovation in health care. Health Aff (Millwood).

[18] Chambers DA, Glasgow RE, Stange KC (2013). The dynamic sustainability framework: addressing the paradox of sustainment amid ongoing change. Implement Sci.

[19] Giger JT, Pope ND, Vogt HB, Gutierrez C, Newland LA, Lemke J, Lawler MJ (2015). Remote patient monitoring acceptance trends among older adults residing in a frontier state. Comput Hum Behav.

[20] Powell BJ, Fernandez ME, Williams NJ, Aarons GA, Beidas RS, Lewis CC, McHugh SM, Weiner BJ (2019). Enhancing the impact of implementation strategies in healthcare: a research agenda. Front Public Health.

[21] Aanestad M, Jensen TB (2011). Building nation-wide information infrastructures in healthcare through modular implementation strategies. J Strateg Inf Syst.

[22] van Dyk L (2014). A review of telehealth service implementation frameworks. Int J Environ Res Public Health.

[23] Eom D, Lee H (2017). A holistic approach to exploring the divided standards landscape in E-Health research. ITU Kaleidoscope: Challenges for a Data-Driven Society.

[24] Larinkari S, Liisanantti JH, Ala-Lääkkölä T, Meriläinen M, Kyngäs H, Ala-Kokko T (2016). Identification of tele-ICU system requirements using a content validity assessment. Int J Med Inform.

[25] Moeckli J, Cram P, Cunningham C, Reisinger HS (2013). Staff acceptance of a telemedicine intensive care unit program: a qualitative study. J Crit Care.

[26] Damschroder LJ, Aron DC, Keith RE, Kirsh SR, Alexander JA, Lowery JC (2009). Fostering implementation of health services research findings into practice: a consolidated framework for advancing implementation science. Implement Sci.

[27] Damschroder LJ, Lowery JC (2013). Evaluation of a large-scale weight management program using the consolidated framework for implementation research (CFIR). Implement Sci.

[28] Gimbel S, Mwanza M, Nisingizwe MP, Michel C, Hirschhorn L, AHI PHIT Partnership Collaborative (2017). Improving data quality across 3 sub-Saharan African countries using the Consolidated Framework for Implementation Research (CFIR): results from the African Health Initiative. BMC Health Serv Res.

[29] Abbott PA, Foster J, de Fatima Marin H, Dykes PC (2014). Complexity and the science of implementation in health IT--knowledge gaps and future visions. Int J Med Inform.

[30] Powell BJ, Waltz TJ, Chinman MJ, Damschroder LJ, Smith JL, Matthieu MM, Proctor EK, Kirchner JE (2015). A refined compilation of implementation strategies: results from the Expert Recommendations for Implementing Change (ERIC) project. Implement Sci.

[31] Waltz TJ, Powell BJ, Matthieu MM, Damschroder LJ, Chinman MJ, Smith JL, Proctor EK, Kirchner JE (2015). Use of concept mapping to characterize relationships among implementation strategies and assess their feasibility and importance: results from the Expert Recommendations for Implementing Change (ERIC) study. Implement Sci.

[32] Damschroder LJ, Powell BJ, Waltz TJ (2015). Expert recommendations for tailoring strategies to context. U.S. Department of Veteran Affairs.

[33] Proctor EK, Powell BJ, McMillen JC (2013). Implementation strategies: recommendations for specifying and reporting. Implement Sci.

[34] Kumar A, Pore P, Gupta S, Wani AO (2016). Level of stress and its determinants among intensive care unit staff. Indian J Occup Environ Med.

[35] Kahn JM (2014). What we talk about when we talk about intensive care unit strain. Ann Am Thorac Soc.

[36] Drew BJ, Harris P, Zègre-Hemsey JK, Mammone T, Schindler D, Salas-Boni R, Bai Y, Tinoco A, Ding Q, Hu X (2014). Insights into the problem of alarm fatigue with physiologic monitor devices: a comprehensive observational study of consecutive intensive care unit patients. PLoS One.

[37] Harrison JP, Harrison JP (2016). Strategic planning and SWOT analysis. Essentials of Strategic Planning in Healthcare.

[38] O'Brien BC, Harris IB, Beckman TJ, Reed DA, Cook DA (2014). Standards for reporting qualitative research: a synthesis of recommendations. Acad Med.

[39] Poncette AS, Meske C, Mosch L, Balzer F (2018). How to overcome barriers for the implementation of new information technologies in intensive care medicine. 20th International Conference on Human Interface and the Management of Information.

[40] Vital SyncTM virtual patient monitoring platform 2.6. Medtronic.

[41] Dubois A, Gadde LE (2002). Systematic combining: an abductive approach to case research. J Bus Res.

[42] Zainal Z (2007). Case study as a research method. J Kemanus.

[43] Poncette AS, Spies C, Mosch L, Schieler M, Weber-Carstens S, Krampe H, Balzer F (2019). Clinical requirements of future patient monitoring in the intensive care unit: qualitative study. JMIR Med Inform.

[44] Neyer FJ, Felber J, Gebhardt C (2012). Entwicklung und Validierung einer Kurzskala zur Erfassung von Technikbereitschaft. Diagnostica.

[45] Harris PA, Taylor R, Thielke R, Payne J, Gonzalez N, Conde JG (2009). Research electronic data capture (REDCap)--a metadata-driven methodology and workflow process for providing translational research informatics support. J Biomed Inform.

[46] Harris PA, Taylor R, Minor BL, Elliott V, Fernandez M, O'Neal L, McLeod L, Delacqua G, Delacqua F, Kirby J, Duda SN, REDCap Consortium (2019). The REDCap consortium: building an international community of software platform partners. J Biomed Inform.

[47] Noble H, Smith J (2015). Issues of validity and reliability in qualitative research. Evid Based Nurs.

[48] Fereday J, Muir-Cochrane E (2006). Demonstrating rigor using thematic analysis: a hybrid approach of inductive and deductive coding and theme development. Int J Qual Methods.

[49] Boyatzis RE (1998). Transforming qualitative information: thematic analysis and code development.

[50] Crabtree BF, Miller WF, Crabtree BF, Miller WL (1992). A template approach to text analysis: developing and using codebooks. Doing qualitative research.

[51] (2019). VERBI software. MAXQDA.

[52] (2016). Strategy design. Consolidated Framework for Implementation Research.

[53] Aref-Adib G, McCloud T, Ross J, O'Hanlon P, Appleton V, Rowe S, Murray E, Johnson S, Lobban F (2019). Factors affecting implementation of digital health interventions for people with psychosis or bipolar disorder, and their family and friends: a systematic review. Lancet Psychiatry.

[54] Labrique AB, Wadhwani C, Williams KA, Lamptey P, Hesp C, Luk R, Aerts A (2018). Best practices in scaling digital health in low and middle income countries. Global Health.

[55] Poncette AS, Mosch L, Spies C, Schmieding M, Schiefenhövel F, Krampe H, Balzer F (2020). Improvements in patient monitoring in the intensive care unit: survey study. J Med Internet Res.

[56] Chochoiek N (2017). Explaining the success of user-centered design - an empirical study across German B2C firms. Jr Manag Sci.

[57] Andrade E, Quinlan LR, Harte R, Byrne D, Fallon E, Kelly M, O'Connor P, O'Hora D, Scully M, Laffey J, Pladys P, Beuchée A, ÓLaighin G (2018). Investigation of the human factors, usability and user experience of patient monitors used in a hospital setting. International Conference on Human Systems Engineering and Design: Future Trends and Applications.

[58] Ross J, Stevenson F, Dack C, Pal K, May C, Michie S, Barnard M, Murray E (2018). Developing an implementation strategy for a digital health intervention: an example in routine healthcare. BMC Health Serv Res.

[59] Leeman J, Birken SA, Powell BJ, Rohweder C, Shea CM (2017). Beyond "implementation strategies": classifying the full range of strategies used in implementation science and practice. Implement Sci.

[60] Shaw J, Agarwal P, Desveaux L, Palma DC, Stamenova V, Jamieson T, Yang R, Bhatia RS, Bhattacharyya O (2018). Beyond "implementation": digital health innovation and service design. NPJ Digit Med.

[61] Halpern NA (2014). Innovative designs for the smart ICU: part 1: from initial thoughts to occupancy. Chest.

[62] Broens S, Dahan A, van Velzen M (2020). Challenges and pitfalls with a randomized clinical trial in the postanesthesia care unit. Sage Res Method Cases.

[63] Koenig MA (2019). Telemedicine in the ICU.

[64] Moullin JC, Sabater-Hernández D, Fernandez-Llimos F, Benrimoj SI (2015). A systematic review of implementation frameworks of innovations in healthcare and resulting generic implementation framework. Health Res Policy Syst.

[65] Ray JM, Ratwani RM, Sinsky CA, Frankel RM, Friedberg MW, Powsner SM, Rosenthal DI, Wachter RM, Melnick ER (2019). Six habits of highly successful health information technology: powerful strategies for design and implementation. J Am Med Inform Assoc.

[66] Halpern NA, Anderson DC, Kesecioglu J (2017). ICU design in 2050: looking into the crystal ball!. Intensive Care Med.

[67] Falini S, Angelotti G, Cecconi M (2020). ICU management based on big data. Curr Opin Anaesthesiol.

[68] Guimarães T, Moreira A, Peixoto H, Santos M (2020). ICU data management - a permissioned blockchain approach. Procedia Comput Sci.

[69] Krohn R, Metcalf D, Salber P (2017). Connected health: improving care, safety, and efficiency with wearables and IoT solution.

[70] Kang M, Park E, Cho BH, Lee KS (2018). Recent patient health monitoring platforms incorporating internet of things-enabled smart devices. Int Neurourol J.

[71] Mahmud R, Koch FL, Buyya R (2018). Cloud-fog interoperability in IoT-enabled healthcare solutions. Proceedings of the 19th International Conference on Distributed Computing and Networking.

[72] Egala BS, Priyanka S, Pradhan AK (2019). SHPI: smart healthcare system for patients in ICU using IoT. IEEE International Conference on Advanced Networks and Telecommunications Systems.

[73] Padhy RP, Patra MR, Satapathy SC (2011). Cloud computing: security issues and research challenges. Int J Comput Sci Inf Technol.

[74] Jaleel A, Mahmood T, Hassan MA, Bano G, Khurshid SK (2020). Towards medical data interoperability through collaboration of healthcare devices. IEEE Access.

[75] Lehne M, Sass J, Essenwanger A, Schepers J, Thun S (2019). Why digital medicine depends on interoperability. NPJ Digit Med.

[76] Kang S, Baek H, Jung E, Hwang H, Yoo S (2019). Survey on the demand for adoption of Internet of Things (IoT)-based services in hospitals: investigation of nurses' perception in a tertiary university hospital. Appl Nurs Res.

[77] Kupwade Patil H, Seshadri R (2014). Big data security and privacy issues in healthcare. 2014 IEEE International Congress on Big Data.

[78] Wilkowska W, Ziefle M (2012). Privacy and data security in E-health: requirements from the user's perspective. Health Informatics J.

[79] Kruse CS, Frederick B, Jacobson T, Monticone DK (2017). Cybersecurity in healthcare: a systematic review of modern threats and trends. Technol Health Care.

[80] Jalali MS, Kaiser JP (2018). Cybersecurity in hospitals: a systematic, organizational perspective. J Med Internet Res.

[81] Topol E (2019). Preparing the healthcare workforce to deliver the digital future. National Health Service.

[82] Luetz A, Grunow JJ, Mörgeli R, Rosenthal M, Weber-Carstens S, Weiss B, Spies C (2019). Innovative ICU solutions to prevent and reduce delirium and post-intensive care unit syndrome. Semin Respir Crit Care Med.

[83] Ince C (2019). Physiology and technology for the ICU in vivo. Crit Care.

[84] Human factors engineering lab - overview. Mayo Clinic.

[85] Bauer MS, Damschroder L, Hagedorn H, Smith J, Kilbourne AM (2015). An introduction to implementation science for the non-specialist. BMC Psychol.

[86] Rycroft-Malone J, Seers K, Chandler J, Hawkes CA, Crichton N, Allen C, Bullock I, Strunin L (2013). The role of evidence, context, and facilitation in an implementation trial: implications for the development of the PARIHS framework. Implement Sci.

[87] Rycroft-Malone J, Kitson A, Harvey G, McCormack B, Seers K, Titchen A, Estabrooks C (2002). Ingredients for change: revisiting a conceptual framework. Qual Saf Health Care.

[88] May C, Finch T, Mair F, Ballini L, Dowrick C, Eccles M, Gask L, MacFarlane A, Murray E, Rapley T, Rogers A, Treweek S, Wallace P, Anderson G, Burns J, Heaven B (2007). Understanding the implementation of complex interventions in health care: the normalization process model. BMC Health Serv Res.

[89] Stetler CB, Damschroder LJ, Helfrich CD, Hagedorn HJ (2011). A guide for applying a revised version of the PARIHS framework for implementation. Implement Sci.

[90] Kukafka R, Johnson SB, Linfante A, Allegrante JP (2003). Grounding a new information technology implementation framework in behavioral science: a systematic analysis of the literature on IT use. J Biomed Inform.

[91] Meyers DC, Durlak JA, Wandersman A (2012). The quality implementation framework: a synthesis of critical steps in the implementation process. Am J Community Psychol.

[92] Mann H, Grant G, Mann IJ (2009). Green IT: an implementation framework. Proceedings of the 15th Americas Conference on Information Systems.

[93] Rosa RG, Teixeira C, Sjoding M (2020). Novel approaches to facilitate the implementation of guidelines in the ICU. J Crit Care.

[94] Holdsworth LM, Safaeinili N, Winget M, Lorenz KA, Lough M, Asch S, Malcolm E (2020). Adapting rapid assessment procedures for implementation research using a team-based approach to analysis: a case example of patient quality and safety interventions in the ICU. Implement Sci.

