# Supplementary material for: Creation of an Evidence-Based Implementation Framework for Digital Health Technology in the Intensive Care Unit: Qualitative Study
Source: JMIR Form Res. 2022 Apr 8;6(4):e22866. doi: 10.2196/22866 (PMC9034425; doi:10.2196/22866)
Supplement: Multimedia Appendix 1 [file formative_v6i4e22866_app1.docx]


**Semi-structured interview**

Datum:____________ Running. No..:______________

**Remote patient monitoring system: CFIR - adapted questionnaire**

| Question | CFIR Category |
| --- | --- |
| **Current patient monitoring in ICU** | |
| 1. I am satisfied with the current patient monitoring system.   Likert scale 1= not correct at all - 5 = completely correct   1. In my opinion, there is a need to change or improve patient monitoring on the ward.   Likert Scale 1= not correct at all - 5 = completely correct | Inner Setting - Implementation Climate: Tension for Change |
| 1. What should be changed?   Graphical representation  Alarm Management  Intuitive operation  Less cables  Interoperability with other devices (ventilator, PDMS, ...)  Trend analyses  Setting advanced functions  Other proposed amendments:  Likert Scale 1= not correct at all - 5 = completely correct | Inner Setting - Implementation Climate: Tension for Change |
| **Remote patient monitoring e.g. via smartphones or tablets in the intensive care unit** | |
| 1. Remote monitoring of patients in the intensive care unit offers advantages.   Likert Scale 1= not correct at all - 5 = completely correct | Inner Setting - Implementation Climate: Compatibility  Intervention characteristics: relative advantage |
| 1. I personally find the implementation of remote patient monitoring in intensive care important.   Likert scale: 1= not correct at all - 5 = completely correct | Inner Setting - Implementation Climate: Relative Priority  Individual Characteristics - knowledge and beliefs about the intervention |
| 1. Why do you find the introduction of remote patient monitoring (not) important? | Inner setting - Implementation Climate: compatibility, relative priority  Individual characteristics - knowledge and beliefs about the intervention |
| **VitalSync remote patient monitoring system** | |
| 1. I find the use of the VitalSync System as a supplement to the current patient monitoring system useful.   Likert scale: 1= not correct at all - 5 = completely correct | Intervention characteristics - relative advantage  Individual characteristics - knowledge and beliefs about the intervention |
| 1. The VitalSync system was of high benefit to my profession.   Likert scale: 1= not correct at all - 5 = completely correct   1. Why (not)? |  |
| 1. Using VitalSync increases patient safety in the intensive care unit.   Likert scale: 1= not correct at all - 5 = completely correct   1. Why (not)? | Outer setting: Patient needs and resources  Individual characteristics - knowledge and beliefs about the intervention |
| 1. In my opinion, the VitalSync System has been well integrated into the daily work on the ward.   Likert scale: 1= not correct at all - 5 = completely correct   1. What do you think was the reason for that? | Intervention Characteristics: Complexity,  Adaptability  Process: Executing |
| **Design** | |
| 1. The design of the VitalSync System is intuitive.   Likert scale: 1= not correct at all - 5 = completely correct   1. The design of the VitalSync System is attractive.   Likert scale: 1= not correct at all - 5 = completely correct   1. The design of the VitalSync System is clear.   Likert scale: 1= not correct at all - 5 = completely correct | IC: Design Quality and Packaging |
| **Communication of the project** | |
| 1. I feel well informed about the process of installing the VitalSync System on the PACU.   Likert scale: 1= not correct at all - 5 = completely correct   1. The purpose and goals of the VitalSync project were clearly communicated by the project leaders.   Likert scale: 1= not correct at all - 5 = completely correct   1. What would you have wished for in terms of communication by the project managers? | Inner Setting: Networks and communication, Intervention source, Implementation Climate: Goals and feedback, Learning climate  Process: Planning, Engaging |
| 1. Please evaluate how far the following factors contribute to the successful implementation of technologies like VitalSync from your perspective.   Likert scale: 1= not correct at all - 5 = completely correct   - 1. High intuitiveness   2. Great additional benefit   3. Low personnel turnover (constant personnel pool, little leasing)   4. Low patient turnover on the ward (longer stays)   5. Reduced workload   6. a lot of technical instructions on the system   7. All patient beds in the ward equipped with the system (extensive availability)   8. Higher number of available parameters in monitoring  1. Other factors that contribute significantly to a successful implementation of the system: | Intervention characteristics: Relative advantage, adaptability, evidence strength and quality  Inner setting: structural characteristics |
| Teamwork on the ward |  |
| 1. I feel well integrated into the interprofessional team in the intensive care unit.   Likert scale: 1= not correct at all - 5 = completely correct   1. The team spirit within the team on the ward is good.   Likert scale: 1= not correct at all - 5 = completely correct   1. The atmosphere within the team had a great influence on the introduction of the VitalSync System on the ward.   Likert scale: 1= not correct at all - 5 = completely correct   1. Please give reasons for your decision. | Inner Setting: Networks and communication  Process: Enganging, Executing |
| **Hierarchical structures on the ward** | |
| 1. Hierarchies, both within and between professions (e.g. within the nursing team / between nurses and doctors) play a major role.   Likert scale: 1= not correct at all - 5 = completely correct   1. Hierarchical structures on the ward had a great influence on the introduction of the VitalSync System.   Likert scale: 1= not correct at all - 5 = completely correct   1. Please give reasons for your decision. | Inner setting Networks and communications  Process: Executing |
| **Adaptation to new situations** | |
| 1. In general, I am able to adapt well to new situations and challenges.   Likert scale: 1= not correct at all - 5 = completely correct | CI: Self efficacy |
| 1. Technology acceptance Neyer et al. | Individual Characteristics: self efficacy |
| **Demographic data** |  |
| 1. Qualification    1. Physician in postgraduate specialization    2. Specialist in anaesthesiology    3. Additional qualification in intensive care medicine    4. Additional qualification in emergency medicine    5. Additional qualification in pain medicine    6. Nurse    7. Specialist nurse for intensive care and anaesthesia    8. Paramedic    9. Respiratory therapist 2. How old are you?    1. 18-24 years    2. 25-34 years    3. 35-44 years    4. 45-54 years    5. 55-64 years    6. >65 years |  |
| 1. Gender    1. female    2. male    3. other    4. not specified 2. I am satisfied with my job.   Likert scale: 1= not correct at all - 5 = completely correct | CI: individual stage of change |

[Technical readiness](https://econtent.hogrefe.com/doi/pdf/10.1026/0012-1924/a000067) according to Ney et al.
