# Supplementary material for: Creation of an Evidence-Based Implementation Framework for Digital Health Technology in the Intensive Care Unit: Qualitative Study
Source: JMIR Form Res. 2022 Apr 8;6(4):e22866. doi: 10.2196/22866 (PMC9034425; doi:10.2196/22866)
Supplement: Multimedia Appendix 2 [file formative_v6i4e22866_app2.docx]

Mapping of CFIR domains to summaries of codes concerning the implementation performances.

| CFIR Construct |  | Summary segment and questionnaire responses |
| --- | --- | --- |
| **Intervention characteristics** |  |  |
|  | *Intervention source* | There was a feeling among the staff that the remote patient monitoring system was being imposed from above without having an influence on being part of the implementation. |
|  | *Evidence strength and quality* | The installed beta version of the system only offered a limited number of vital parameters that could be monitored. |
|  | *Relative advantage* | The system was introduced to the ICU as a supplementary monitoring system, which was why the staff did not perceive its additional value as high. |
|  |  | The current monitoring system already offered remote monitoring functions such as flexibly, displaying parameters of different patients on a bedside monitor |
|  |  | In an ICU, the reaction to an alarm has to be immediate due to the severe conditions of the admitted patients. |
|  |  | Remotely monitoring patients while being on a different ward or in a different part of the hospital makes the quick reaction impossible and, thus, is without consequence. |
|  |  | The available vital signs were not sufficient to evaluate the patient's condition satisfactorily. |
|  |  | The majority stated that using the remote patient monitoring as a supplementary monitoring in the ICU was not useful (5 not correct at all or not quite correct), did not have a benefit for the respective professional group (5 not correct at all or not quite correct,) [...]. |
|  | *Trialability* | The system was introduced to the ICU as a supplementary monitoring system, which was why the staff did not perceive its additional value as high. |
|  | *Adaptability* | The system was introduced to the ICU as a supplementary monitoring system, which was why the staff did not perceive its additional value as high. |
|  |  | In an ICU, the reaction to an alarm has to be immediate due to the severe conditions of the admitted patients. |
|  |  | Remotely monitoring patients while being on a different ward or in a different part of the hospital makes the quick reaction impossible and, thus, is without consequence. |
|  |  | If physicians are registering a remote alarm while being occupied with tasks such as placing a central venous catheter or performing surgery, those alarms remain without consequence. |
|  |  | In key situations such as transport of patients, using the system for monitoring was not possible due to a large module and several cables. |
|  |  | The majority stated that [...] the remote patient monitoring as a supplementary monitoring in the ICU [...] was not well integrated into the clinical routine (5 not correct at all or not quite correct). |
|  | *Complexity* | The high patient turnover in a post-anesthesia care unit led to a higher workload for nursing staff while connecting and disconnecting the system for every new patient. |
|  |  | The tablet was perceived by some interviewees as too large for using it in the daily work routine. |
|  |  | A reason for not using the system was the inconvenience of carrying another device in already packed tunic pockets. |
|  |  | A concern was that the device can get lost. |
|  |  | Interviewees raised concerns about the system's dependency on a stable WiFi connection. |
| **Inner setting** |  |  |
|  | *Structural characteristics* | Training did not reach all staff due to a complex shift system and a big pool of staff for two ICUs. |
|  |  | The system was implemented only at five out of ten bedsides on one out of two ICUs. |
|  |  | The high number of monitors in an ICU made an additional tablet not necessary, according to the interviewees. |
|  |  | The perceived impact of the system was low because high staff presence in the ICU made the implementation of remote patient monitoring superfluous. |
|  |  | The high patient turnover in a post-anesthesia care unit led to a higher workload for nursing staff while connecting and disconnecting the system for every new patient. |
|  |  | Respondents claimed that in an ICU, the reaction to an alarm has to be immediate due to the severe conditions of the admitted patients. Remotely monitoring patients while being on a different ward or in a different part of the hospital makes the quick reaction impossible and, thus, is without consequence. |
|  | *Networks and communications* | There was a lack of persisting initiatives to engage staff in the implementation process such as regular staff training and information events. |
|  |  | Communication of negative aspects of the intervention created a negative peer pressure to not use the system. |
|  |  | Training did not reach all staff due to a complex shift system and a big pool of staff for two ICUs. |
|  |  | The team spirit on the ICU was stated to be good by the majority (7 quite correct). |
|  |  | The general atmosphere within the ICU team had a large impact on the implementation process according to the interviewees (5 quite correct). |
|  |  | The majority stated that the aims and purpose of the remote patient monitoring implementation was clearly communicated (6 quite correct or completely correct ). |
|  | *Implementation climate*  - Tension for change | The system was introduced to the ICU as a supplementary monitoring system, which was why the staff did not perceive its additional value as high. |
|  |  | The current monitoring system already offered remote monitoring functions, such as flexibly displaying parameters of different patients on a bedside monitor. |
|  |  | ICU staff saw no additional benefit in using the system. |
|  |  | Interviewees said to be satisfied with the current patient monitoring and do not see the need for change. |
|  | *Implementation climate*  - Compatibility | Staff involvement was perceived to be more targeted towards nursing staff, although not being in charge of the implementation project. |
|  | *Implementation climate*  - Relative priority | There was a lack of feeling of responsibility by staff members of all professions to continuously apply the system. |
|  | *Implementation climate*  - Learning climate | On the one hand, a leading nurse or physician was not identified for the implementation process by the staff, on the other hand, interviewees reported a lack of persisting initiatives to engage staff in the implementation process such as regular training and information events. |
|  |  | The high patient turnover in a post-anesthesia care unit led to a higher workload for nursing staff while connecting and disconnecting the system for every new patient. |
|  | *Implementation readiness*  - Leadership engagement | A lack of leadership engagement was reported both in the nursing and the medical department. |
|  |  | Staff could not identify a leading nurse or physician responsible for the implementation process. |
|  | *Implementation readiness*  - access to information and knowledge | A lack of knowledge about the aims and context of the implementation project, and a lack of continuous staff training led to a negative feeling towards the intervention, and a lack of motivation to engage further with the system. |
| **Individual characteristics** |  |  |
|  | *Knowledge and beliefs about the intervention* | Communication of negative aspects of the intervention created a negative peer pressure to not use the system. |
|  |  | A lack of knowledge about the aims and context of the implementation project, and a lack of continuous staff training led to a negative feeling towards the intervention and a lack of motivation to engage further with the system. |
|  |  | The staff was afraid of losing their break times when applying the remote patient monitoring system. |
|  |  | Another fear was that the system creates an increased workload for staff (e.g., set-up, connection) rather than decreasing it. |
|  |  | ICU staff claimed that remote monitoring would lead to less direct patient contact and thus threaten patient safety as the patient's clinical condition would not be evaluated adequately. |
|  |  | The staff feared that additional (false) alarms would appear when applying the system, increasing the stress level and endangering patient safety. |
|  | *Self-efficacy* | Interviewees accomplished, on average, 47 points on a 12–60 point technology commitment scale, which shows high technology commitment. |
|  |  | ICU staff did not use the system because they lacked the habit and routine to use remote patient monitoring technology. |
|  | *Individual stage of change* | ICU staff saw no additional benefit in using the system. |
|  |  | Opinions were split if patient monitoring improvements are necessary (3 not correct at all or not quite correct, 4 quite correct or completely correct ), if remote patient monitoring of patients in the ICU has advantages (1 not quite correct, 3 quite correct or completely correct ), and if remote patient monitoring in the ICU is important (2 not quite correct, 1 completely correct). |
|  |  | The majority of participants stated to be satisfied with the current patient monitoring (6 quite correct or completely correct). |
| **Process** |  |  |
|  | *Planning* | There was a lack of knowledge among staff about the aims and context of the implementation project. |
|  | *Engaging* | Staff involvement was perceived to be more targeted towards nursing staff, although not being in charge of the implementation project. |
|  |  | There was a lack of persisting initiatives to engage staff in the implementation process such as regular staff training and information events. |
|  | *Engaging*  - Opinion leaders  - Peers | Communication of negative aspects of the intervention created a negative peer pressure to not use the system. |
|  | *Engaging*  - Opinion leaders  - Experts | Physicians were perceived to be not as engaged with the system and to have received less training. |
|  | *Engaging*  - Formally appointed internal implementation leaders | Staff could not identify a leading nurse or physician responsible for the implementation process. |
|  | *Executing* | The frequency of staff training was very high in the beginning but decreased over time. Staff said to have felt well informed about the project initially, however, the information flow decreased equally. |
