# Supplementary material for: Creation of an Evidence-Based Implementation Framework for Digital Health Technology in the Intensive Care Unit: Qualitative Study
Source: JMIR Form Res. 2022 Apr 8;6(4):e22866. doi: 10.2196/22866 (PMC9034425; doi:10.2196/22866)
Supplement: Multimedia Appendix 3 [file formative_v6i4e22866_app3.docx]

ERIC strategies mapped to summaries of codes concerning staff suggestions for improving implementation performance.

| ERIC strategy |  | Summary segment and questionnaire responses |
| --- | --- | --- |
| **Use evaluative and iterative strategies** |  |  |
|  | *Purposely re-examine the implementation* | Feedback discussions with staff and project leaders during implementation would increase staff engagement. |
|  | *Develop a formal implementation blueprint* | Staff should be informed of the implementation project and its aims in order to increase motivation to apply the new technology. |
|  | *Audit and provide feedback* | Feedback discussions with staff and project leaders during implementation would increase staff engagement. |
| **Provide interactive assistance** |  |  |
|  | *Facilitation* | During training, staff should be informed of the implementation project and its aims in order to increase motivation to apply the new technology. |
|  |  | A well-functioning team with good team spirit was deemed beneficial for successful implementation. |
|  | *Provide clinical supervision* | Persistent leadership engagement and the nomination of specific responsible persons for the implementation process was important for successful implementation, especially in a busy environment such as the ICU. |
| **Adapt and tailor to context** |  |  |
|  | *Promote adaptability* | During training, staff should be informed of the implementation project and its aims in order to increase motivation to apply the new technology. |
|  |  | With regard to the Software of the monitoring technology, interoperability with other devices such as the respirator or the PDMS is important for a successful implementation of a remote patient monitoring system, especially regarding import and export of patient data and visualization of parameters on one screen. |
|  |  | High intuitiveness is crucial for effective implementation. |
|  |  | A large screen for clear visualization is demanded, on the other hand interviewees favored a device that fits into the pocket of a tunic, e.g. a smartphone. |
|  |  | The intelligent grouping and display of monitoring parameters thematically by organ systems is advocated. Visualization of alarms should be clearer. Intelligent alarm management would be beneficial. Remote patient monitoring via smartphone could work well with vibration alarms. |
|  |  | Availability of all standard vital sign parameters (7 quite correct or completely correct), high intuitiveness (6 quite correct or completely correct) and high additional benefit (6 quite correct or completely correct) of the intervention would facilitate implementation. |
| **Develop stakeholder interrelationships** |  |  |
|  | *Recruit, designate, and train for leadership* | Persistent leadership engagement and the nomination of specific responsible persons for the implementation process was important for successful implementation, especially in a busy environment such as the ICU. |
|  |  | Furthermore, communication of the project by team leaders and coordinators should be encouraging and motivating. |
|  | *Organize clinician implementation team meetings* | Feedback discussions with staff and project leaders during implementation would increase staff engagement. |
|  | *Model and simulate change* | Staff should be informed of the implementation project and its aims in order to increase motivation to apply the new technology |
|  | *Involve executive boards* | Persistent leadership engagement and the nomination of specific responsible persons for the implementation process was important for successful implementation, especially in a busy environment such as the ICU. |
|  | *Inform local opinion leaders* | Persistent leadership engagement and the nomination of specific responsible persons for the implementation process was important for successful implementation, especially in a busy environment such as the ICU. |
|  |  | Feedback discussions with staff and project leaders during implementation would increase staff engagement. |
|  | *Identify and prepare champions* | Persistent leadership engagement and the nomination of specific responsible persons for the implementation process was important for successful implementation, especially in a busy environment such as the ICU. |
|  |  | Furthermore, communication of the project by team leaders and coordinators should be encouraging and motivating. |
| **Train and educate stakeholders** |  |  |
|  | *Conduct ongoing training* | Furthermore, staff training should take place continuously before or after shifts and was particularly important in early implementation stages. |
|  |  | High frequency of staff training would increase implementation success (5 quite correct or completely correct, 1 not quite correct). |
|  | *Make training dynamic* | The quality of the instructions was essential to positively influence the staff's opinion towards the implementation. |
|  |  | Furthermore, communication of the project by team leaders and coordinators should be encouraging and motivating. |
|  | *Conduct educational meetings* | The personnel should be informed of the implementation project and its aims in order to increase motivation to apply the new technology. |
| **Support clinicians** |  |  |
|  | *Remind clinicians* | Furthermore, staff training should take place continuously before or after shifts and was particularly important in early implementation stages. High frequency of staff training increased implementation success. During training, staff should be informed of the implementation project and its aims in order to increase motivation to apply the new technology. The quality of the instructions was essential to positively influence the staff's opinion towards the implementation. Feedback discussions with staff and project leaders during implementation would increase staff engagement. |
|  | *Facilitate relay of clinical data to providers* | During training, staff should be informed of the implementation project and its aims in order to increase motivation to apply the new technology. The quality of the instructions was essential to positively influence the staff's opinion towards the implementation. Feedback discussions with staff and project leaders during implementation would increase staff engagement. |
| **Change infrastructure** |  |  |
|  | *Change service sites* | A normal ward or IMCU would be more suitable for a remote patient monitoring technology, as staff presence is lower and technical facilities are scarcer. Patients with a relatively weak indication for admission to the intensive care unit, such as postoperative monitoring in a patient with sleep apnea, could thus be admitted to normal ward or IMCU. |
|  |  | Wards with low staff turnover (6 quite correct or completely correct) and low patient turnover (5 quite correct or completely correct) would be more suitable according to the majority. |
|  | *Change physical structure and equipment* | To increase implementation performance, all beds on the ward should be equipped with the respective technology, equally all staff members should have access to a portable monitoring device (tablet, smartphone). |
|  |  | The majority stated ubiquitous availability of the technology (all beds equipped) would facilitate implementation (7 quite correct or completely correct). |
